# A Phase II Study of Docetaxel in Patients with Relapsed and Refractory Ewing's Tumours

**DOI:** 10.1080/1357714031000114192

**Published:** 2003-03

**Authors:** T. Meyer, A. Mctiernan, J. Whelan

**Affiliations:** The London Bone and Soft Tissue Tumour Service The Meyerstein Institute of Oncology University College London Hospitals London W1N 8AA UK

## Abstract

*Purpose*. The prognosis for patients with Ewing's tumours who have metastases at presentation or who are refractory to standard chemotherapy regimens remains poor. There is therefore a need to evaluate the role of new agents. This report describes the initial results of a prospective phase II trial of docetaxel in patients with progressive or refractory Ewing's tumours.

*Patients and methods*. Fourteen patients with Ewing's tumours who had all relapsed or progressed after treatment with multi-drug cytotoxic therapy were treated with docetaxel 100 mg/m^2^ infused over 1 h, three weekly for a maximum of six cycles. Nine patients received granulocyte colony-stimulating factor with all cycles.

*Results*. A partial response was observed in one patient and stable disease in two. The remaining patients progressed on treatment. The major toxicity was myelosuppression and infection with 36% patients experiencing grade 3 or 4 neutropenia and/or infection.

*Conclusion*. Docetaxel appears to have some activity in Ewing's tumours even in heavily pre-treated patients. Further evaluation of its efficacy at an earlier stage of the disease is warranted.


*Sarcoma* (2003) 7, 13–17

Taylor & Francis healthsciences

ORIGINAL ARTICLE

### A phase II study of docetaxel in patients with relapsed and refractory Ewing's Tumours

T. MEYER, A. MCTIERNAN & J. WHELAN

*The London Bone and Soft Tissue Tumour Service, The Meyerstein Institute of Oncology, University College London Hospitals, London W1N 8AA, UK*

**Abstract**
*Purpose.* The prognosis for patients with Ewing's tumours who have metastases at presentation or who are refractory to standard chemotherapy regimens remains poor. There is therefore a need to evaluate the role of new agents. This report describes the initial results of a prospective phase II trial of docetaxel in patients with progressive or refractory Ewing's tumours.
*Patients and methods.* Fourteen patients with Ewing's tumours who had all relapsed or progressed after treatment with multi-drug cytotoxic therapy were treated with docetaxel 100 mg/m$^2$ infused over 1 h, three weekly for a maximum of six cycles. Nine patients received granulocyte colony-stimulating factor with all cycles.
*Results.* A partial response was observed in one patient and stable disease in two. The remaining patients progressed on treatment. The major toxicity was myelosuppression and infection with 36% patients experiencing grade 3 or 4 neutropenia and/or infection.
*Conclusion.* Docetaxel appears to have some activity in Ewing's tumours even in heavily pre-treated patients. Further evaluation of its efficacy at an earlier stage of the disease is warranted.

**Key words:** *Taxotere, bone tumours, taxanes, PNET*

### Introduction

The Ewing's family of tumours includes Ewing's sarcoma of bone and extraosseous sites, primitive neuroectodermal tumours (PNET) and Askin's tumours. All are characterised morphologically as small blue round malignant cells which appear to derive from the same primordial stem cell, differing only in the degree of neural differentiation.[1] These tumours occur most commonly in the second decade of life and have an annual incidence of 0.6/million population.[2]

Approximately 20–30% of patients have overt metastases at presentation, yet, before the introduction of systemic chemotherapy, 90% ultimately died from metastatic disease.[3] With aggressive cytotoxic chemotherapy survival rates of up to 70% are now reported in localised disease.[4,5] indicating both that the tumour is chemosensitive and that it should be regarded as a systemic disease. Standard treatment now comprises multi-drug chemotherapy including agents such as vincristine, ifosfamide, cyclophosphamide, etoposide, actinomycin D and doxorubicin combined with either radiotherapy or surgery or both to the primary site.[5–7] In metastatic disease the prognosis is less favourable with disease-free survival rates ranging between 10 and 20%.[8,9] The site of metastatic disease is an important determinant and those with pulmonary disease have a more favourable outlook than those with bone or bone marrow disease, while those with both have a cure rate of less than 15%.[10] In this poor prognostic group the role of high dose chemotherapy has shown some promise[11] and is being further evaluated in prospective randomised trials.

Recurrent or progressive disease carries a very grave prognosis, particularly if progression occurs on treatment or after a short disease-free interval. In this setting, evaluation of new agents in a phase II study is warranted.

Docetaxel is a semi-synthetic drug derived from a precursor extracted from the needles of *Taxus baccata*.[12] In common with other taxanes such as paclitaxel, docetaxel promotes microtubule assembly and inhibits disassembly thereby causing cellular growth arrest. This class of drug has shown useful activity in a variety of epithelial solid tumours including breast[13–15]and ovarian cancer.[16,17] However, response rates in soft tissue sarcoma and bone tumours have been generally disappointing[18–20] even when used as first line treatment.[21] Ewing's tumours are relatively chemosensitive by comparison

Correspondence to: Dr. J. Whelan. Tel.: +44-207-636-8333, ext 9092; Fax: +44-207-436-0160; E-mail: jeremy.whelan@uclh.org

ISSN 1357-714X print/ISSN 1369-1643 online/03/010013–05 © 2003Taylor & Francis Ltd
DOI: 10.1080/1357714031000114192

with other sarcomas yet no study has specifically examined their response to taxanes. We have therefore performed a prospective phase II study to determine the clinical efficacy and toxicity of docetaxel in relapsed or refractory Ewing's tumours.

### Patients and methods

### *Eligibility and evaluation criteria*

Patients between the ages of 14 and 70 years of age with histologically proven Ewing's tumours were enrolled prospectively into this study, which had been approved by the local ethics committee. All patients had received 'standard' chemotherapy and either progressed or relapsed. Further eligibility criteria included clinically or radiologically assessable disease (measurable in two dimensions) or evaluable disease (measurable in one dimension), WHO performance status $\leq 3$ and a life expectancy greater than 8 weeks. Blood laboratory requirements at entry included an absolute neutrophil count of $\geq 1.5 \times 10^9$/l, platelets $\geq 100 \times 10^9$/l, serum bilirubin $\leq 1 \times$ upper normal limit (UNL), AST and/or ALT $\leq 1.5 \times$ UNL, alkaline phosphatase $\leq 2.5 \times$ UNL (unless bone metastases were present in the absence of liver metastases) and a serum creatinine $\leq 1.5 \times$ UNL. Other exclusion criteria included symptomatic peripheral neuropathy $\geq$ grade 2 by NCI common toxicity criteria, a history of severe hypersensitivity to polysorbate 80 or contraindication to the use of steroids.

Patients provided written consent according to the local ethics committee requirements and, in the case of minors under the age of 18, consent was provided both by the child and the parents.

Prior to entry a full medical history and physical examination was performed including an assessment of performance status, residual toxicity following prior treatments and clinical tumour measurements. The relevant blood tests were also performed, as were a chest X-ray, a technetium bone scan, a pulmonary CT scan and, if appropriate, imaging of the primary site by plain X-ray and CT or MRI scan.

Prior to each treatment the clinical history was recorded including symptoms and toxicities following the previous treatment, weight and performance status. Serum bilirubin, AST, ALT and alkaline phosphatase were analysed before each cycle of treatment and full blood count was performed before treatment and on days 8 and 15 after cycles 1 and 2. Imaging of the primary tumour was performed after every two cycles of docetaxel to assess response. Following completion of therapy patients were followed at regular intervals as clinically indicated and date of progression and/or death recorded.

### *Treatment plan*

Docetaxel was given on an outpatient basis at a dose of 100 mg/m$^2$ by intravenous infusion over 1 h every 21 days for a maximum of six cycles. Dexamethasone was given as pre-medication, 8 mg twice daily starting 24 h before the docetaxel infusion and continuing for a total of 5 days. When the degree of myelosuppression became apparent in this group of heavily pre-treated patients and those in a parallel phase II study in osteosarcoma, growth factors (GCSF, Lenograstim 236 μg/day for 10 days) were used to avoid infection and dose reduction.

Toxicity was assessed according to the National Cancer Institute common toxicity criteria and the appropriate dose modifications made where indicated. The lowest dose allowed following dose reduction was 55 mg/m$^2$.

### *Assessments of clinical response*

A complete response was defined as the complete disappearance of all previously measured tumour for a period of at least 4 weeks. A partial response (PR) was defined as a 50% reduction or more in the sum of the products of two perpendicular diameters of all measured lesions lasting for at least 4 weeks. Progressive disease was recorded if there was an increase in any measurable lesion by 25% or the appearance of a new lesion. Stable disease was defined as a response less than PR or the absence of progression. The duration of response spanned the time of response to disease progression. Time to progression and overall survival were measured from the time of study entry to the time of progression and death.

### Results

### *Patient characteristics*

Fourteen patients aged between 15 and 37 years (median 23.5) were enrolled on the study between April 1997 and December 1999 (Table 1). Five patients had metastases at presentation and all patients initially received prolonged multi-drug chemotherapy in addition to local treatment with surgery and/or radiotherapy. At relapse or progression all but one had pulmonary disease, four had local relapse and two had bone disease. Six patients had at least one trial of further chemotherapy before receiving docetaxel. The median treatment-free interval between the last cycle of chemotherapy and the first cycle of docetaxel was 6 months (range 1–16).

### *Response evaluation*

A total of 40 cycles of docetaxel was given (median number of cycles per patient was 2, range 1–6). Treatment was given at full dose in all patients except patient 1 who received 75% dose after the first cycle because of neutropenic sepsis. Overall there

**Table 1.** *Patient characteristics, treatment and response to docetaxel*

| Patient no. | Age (years) | Primary site | Metastasis | Primary chemotherapy | Local therapy | Event-free-survival* | Site of metastasis | Relapse treatment before docetaxel | Pre-docetaxel free interval months | No. cycles docetaxel given | Response |
|---|---|---|---|---|---|---|---|---|---|---|---|
| 1 | 25 | Scalp | None | VAIA × 7 | RT | 18 (1) | Local and lung | 1. HD-Ifos × 1<br>2. Carbo/Cyclo/Etop × 1<br>3. Dox × 4 | 2 | 5 | PD |
| 2 | 21 | Tibia | None | EVAIA × 13 | EPR+RT | 12 (1) | Local and lung | 1. HD-ifos × 3<br>2. Cyclo<br>Priming and HDT | 5 | 2 | PD |
| 3 | 37 | Sacrum | None | VACA × 12 | RT | 23 (1) | Lung | 1. EVAIA × 2<br>2. HD-ifos × 2 | 2 | 6 | SD |
| 4 | 22 | Pelvis | Lung | P6<br>HDT | RT | 16 (1) | Lung | None | 8 | 3 | PD |
| 5 | 29 | Thigh (extra-osseous) | None | IVAD × 6<br>HDT | EPR | 33 (1) | Lung | EVAIA × 3<br>RT whole ling | 9 | 6 | PR |
| 6 | 20 | Scapular | None | EVAIA × 6 | Excision | 5 (2) | Lung | HD-ifos × 2 | 1 | 3 | PD |
| 7 | 27 | Talus | Bone/lung/ Bone marrow | P6<br>VAIA × 4 | RT | 14 (2) | Lung | None | 6 | 1 | PD |
| 8 | 15 | Pubic ramus | None | EVAIA × 14 | EPR and RT | 18 (1) | Local and bone | None | 9 | 1 | PD |
| 9 | 33 | Femur | None | EVAIA × 14 | EPR | 17 (1) | Lung and bone | HD-ifos × 2 | 1 | 1 | PD |
| 10 | 16 | Fibula | None | EVAIA × 14 | Excision and RT | 11 (1) | Lung | None | 6 | 2 | PD |
| 11 | 27 | Chest wall | Lung | EVAIA × 12 | Excision | 9 (2) | Lung | None | 1 | 1 | PD |
| 12 | 19 | Tibia | Bone | EVAIA × 10<br>HDT | EPR | 24 (1) | Lung | None | 16 | 2 | PD |
| 13 | 19 | Humerus | None | EVAIA × 9<br>VIDE × 3 | Excision | 7 (2) | Local and lung | RT and amputation | 7 | 1 | PD |
| 14 | 27 | Tibia | Lung | VIDE × 6 VIA × 3<br>HD-ifos × 1<br>HDT | Amputation | 18 (1) | Lung | None | 10 | 6 | SD |

*Event free survival: time from start of initial treatment to first relapse (1) or progression (2) CR, complete response; PR, partial response; SD, stable disease; EVAIA, etoposide, vincristine, doxorubicin, ifosfamide, actinomycin D; HDT, busulphan and melphalan with stem cell rescue; RT, radiotherapy; VACA, vincristine, doxorubicin, cyclophosphamide, actinomycin D; P6, alternating regimen containing doxorubicin, cyclophosphamide, vincristinc, etoposide, ifosfamide; VIDE, vincristine, ifosfamide, doxorubicin, etoposide; EPR, endoprosthetic replacement; HD-ifos, ifosfamide 15–18 $g/m^2$.

was one partial response and this was in a patient with extra-osseous Ewing's sarcoma of the thigh. Two patients had stable disease and 11 had disease progression (Table 1). The two patients with stable disease had a progression-free interval of 7 and 10 months, while the partial response was sustained for 2 months. The median survival of all patients was 5 months (range 2–20).

### *Toxicity evaluation*

The major toxicity was myelosuppression, with five patients (36%) experiencing grade 3 or 4 neutropenia and five (36%) experiencing grade 3 infection (Table 2). GCSF was given in 27 out of 41 cycles of chemotherapy (66%). Nine patients who had previously received high dose chemotherapy or multiple previous courses of chemotherapy were treated with GCSF from cycle one onwards. Grade 3 or 4 neutropenia was recorded in six of 27 (22%) cycles in which GCSF was given and in two of 14 (14%) cycles in which it was not. Mild anaemia was also common with 11 (79%) patients developing grade 1 or 2 anaemia and one patient developing grade 3 anaemia. Thrombocytopenia only occurred in three patients. One patient experienced grade 3 stomatitis but this was the only non-haematological toxicity greater than grade 2. Other toxicities recorded at grade 1 and 2 included arthralgia (42%), stomatitis (35%), myalgia and nausea (both 28%), lethargy and rash (both 21%) and neuropathy, constipation and headache (all 14%). There were no episodes of vomiting or anaphylaxis and no toxic deaths. Furthermore, no patient withdrew from study because of toxicity and out of the 40 cycles given only seven were delayed for a median of 7 days (range 1–15).

**Table 2.** *Treatment-related toxicity*

| Toxicity | Patients with toxicity grade | | | |
|---|---|---|---|---|
| | 1 | 2 | 3 | 4 |
| White blood count | 2 | 3 | 3 | 2 |
| Neutropenia | 2 | 2 | 1 | 4 |
| Anaemia | 7 | 4 | 1 | |
| Thrombocytopenia | | 2 | 1 | |
| Arthralgia | 3 | 3 | | |
| Lethargy | 1 | 2 | | |
| Myalgia | 2 | 2 | | |
| Nausea | 4 | | | |
| Stomatitis | 3 | 1 | 1 | |
| Infection | | 7 | 5 | |
| Rash | 2 | 1 | | |
| Neuropathy | 2 | | | |
| Constipation | 2 | | | |
| Headache | | 2 | | |

The worst grade of toxicity experienced for a given side effect during the entire course of chemotherapy was recorded for each patient and the number of patients reporting this grade is shown in the table.

### Discussion

In this study we have evaluated the efficacy and toxicity of docetaxel in patients with Ewing's tumours that have progressed or recurred after prior treatment. Docetaxel was given according to the standard dose and schedule used effectively in other tumours.[13] The patients had all been heavily pre-treated with multi-drug chemotherapy and four patients had received high dose myeloablative chemotherapy as part of their primary treatment, while a further patient received high dose treatment as part of relapse therapy. Furthermore six patients had received at least one trial of relapse chemotherapy. In short, this was a very heavily pre-treated group. In spite of this, one patient achieved a partial response and two had disease stabilisation. As anticipated, myelosuppression was the major toxicity, but the drug was otherwise well tolerated and no patient withdrew from the study because of unacceptable toxicity.

There are very few published data examining the efficacy of docetaxel in Ewing's tumours. In a phase I trial, performed in children with a variety of refractory solid tumours, one partial response and two minimal responses were seen in three patients with peripheral primitive neuroectodermal tumours.[22] Our study therefore represents the first systematic analysis of docetaxel in this rare disease. The number of patients treated is small and insufficient to provide an accurate response rate, but it is unlikely that docetaxel has a role in heavily pre-treated patients. Nevertheless, its activity needs to be further defined at an earlier stage in the disease. Many drugs have been found to be effective as first line treatments while showing little activity in heavily pre-treated patients. Topotecan, for example was ineffective when used in pre-treated patients with rhabdomyosarcoma[23] but gave response rates of 45% as first line treatment.[24] If docetaxel has a role in Ewing's tumours it is likely to be as part of sequential or multi-drug chemotherapy. Data regarding its use in multi-drug regimens is limited, but it has been safely combined with other drugs used in the treatment of Ewings tumours such as doxorubicin,[25,26] suggesting that such combinations are feasible.

Other investigators have reported myelosuppression as the major toxicity of docetaxel and some have suggested that the dose should be lowered to 75 $mg/m^2$.[27] However, the use of GCSF in our group of patients allowed us to maintain full dose treatment in all but one patient and we would recommend a dose of 100 $mg/m^2$ for further studies.

In summary, docetaxel appears to be reasonably tolerated and have some activity in Ewing's tumours. Its role needs to be further evaluated earlier in the disease to determine its efficacy in less heavily pre-treated patients. A phase II window study in patients presenting with metastatic disease would be an appropriate setting to pursue this question.

### References

1. Delattre O, Zucman J, Melot T, *et al.* The Ewing family of tumors, a subgroup of small-round-cell tumors defined by specific chimeric transcripts. *New Engl J Med* 1994; 331: 294–9.
2. Price CH, Jeffree GM. Incidence of bone sarcoma in south west England, 1946–74 in relation to age, sex, tumour site and histology. *Br J Cancer* 1977; 36: 511–22.
3. Patricio MB, Vilhena M, Neves M, *et al.* Ewing's sarcoma in children: twenty-five years of experience at the Instituto Portuges de Oncologia de Francisco Gentil (I.P.O.F.G.). *J Surg Oncol* 1991; 47: 37–40.
4. Bacci G, Ferrari S, Bertoni F, *et al.* Neoadjuvant chemotherapy for peripheral malignant neuroectodermal tumor of bone: recent experience at the istituto rizzoli. *J Clin Oncol* 2000; 18: 885–92.
5. Craft A, Cotterill S, Malcolm A, *et al.* Ifosfamide-containing chemotherapy in Ewing's sarcoma: the Second United Kingdom Children's Cancer Study Group and the Medical Research Council Ewing's Tumor Study. *J Clin Oncol* 1998; 16: 3628–33.
6. Burgert EOJ, Nesbit ME, Garnsey LA, *et al.* Multimodal therapy for the management of nonpelvic, localized Ewing's sarcoma of bone: intergroup study IESS-II . *J Clin Oncol* 1990; 8: 1514–24.
7. Wexler LH, DeLaney TF, Tsokos M, *et al.* Ifosfamide and etoposide plus vincristine, doxorubicin, and cyclophosphamide for newly diagnosed Ewing's sarcoma family of tumors. *Cancer* 1996; 78: 901–11.
8. Cangir A, Vietti TJ, Gehan EA, *et al.* Ewing's sarcoma metastatic at diagnosis. Results and comparisons of two intergroup Ewing's sarcoma studies. *Cancer* 1990; 66: 887–93.
9. Paulussen M, Ahrens S, Craft AW, *et al.* Ewing's tumors with primary lung metastases: survival analysis of 114 (European Intergroup) Cooperative Ewing's Sarcoma Studies patients. *J Clin Oncol* 1998; 16: 3044–52.
10. Paulussen M, Ahrens S, Burdach S, *et al.* Primary metastatic (stage IV) Ewing tumor: survival analysis of 171 patients from the EICESS studies. European Intergroup Cooperative Ewing Sarcoma Studies. *Ann Oncol* 1998; 9: 275–81.
11. Burdach S, Jurgens H, Peters C, *et al.* Myeloablative radiochemotherapy and hematopoietic stem-cell rescue in poor-prognosis Ewing's sarcoma. *J Clin Oncol* 1993; 11: 1482–8.
12. Ringel I, Horwitz SB. Studies with RP 56976 (taxotere): a semisynthetic analogue of taxol. *J Natl Cancer Inst* 1991; 83: 288–91.
13. Chan S, Friedrichs K, Noel D, *et al.* Prospective randomized trial of docetaxel versus doxorubicin in patients with metastatic breast cancer. The 303 Study Group. *J Clin Oncol* 1999; 17: 2341–54.
14. Ferraresi V, Milella M, Vaccaro A, *et al.* Toxicity and activity of docetaxel in anthracycline-pretreated breast cancer patients: a phase II study. *Am J Clin Oncol* 2000; 23: 132–9.
15. Nabholtz JM, Senn HJ, Bezwoda WR, *et al.* Prospective randomized trial of docetaxel versus mitomycin plus vinblastine in patients with metastatic breast cancer progressing despite previous anthracycline-containing chemotherapy. 304 Study Group. *J Clin Oncol* 1999; 17: 1413–24.
16. Gore ME, Levy V, Rustin G, *et al.* Paclitaxel (Taxol) in relapsed and refractory ovarian cancer: the UK and Eire experience. *Br J Cancer* 1995; 72: 1016–9.
17. Thigpen JT, Blessing JA, Ball H, *et al.* Phase II trial of paclitaxel in patients with progressive ovarian carcinoma after platinum-based chemotherapy: a Gynecologic Oncology Group study. *J Clin Oncol* 1994; 12: 1748–53.
18. Edmonson JH, Ebbert LP, Nascimento AG, *et al.* Phase II study of docetaxel in advanced soft tissue sarcomas. *Am J Clin Oncol* 1996; 19: 574–6.
19. van Hoesel QG, Verweij J, Catimel G, *et al.* Phase II study with docetaxel (Taxotere) in advanced soft tissue sarcomas of the adult. EORTC Soft Tissue and Bone Sarcoma Group. *Ann Oncol* 1994; 5: 539–42.
20. Verweij J, Catimel G, Sulkes A, *et al.* Phase II studies of docetaxel in the treatment of various solid tumours. EORTC Early Clinical Trials Group and the EORTC Soft Tissue and Bone Sarcoma Group. *Eur J Cancer* 1995; 31A (Suppl 4): S21–4.
21. Verweij J, Lee SM, Ruka W, *et al.* Randomized phase II study of docetaxel versus doxorubicin in first- and second-line chemotherapy for locally advanced or metastatic soft tissue sarcomas in adults: a study of the European Organization for Research and Treatment of Cancer Soft Tissue and Bone Sarcoma Group. *J Clin Oncol* 2000; 18 :2081–6.
22. Blaney SM, Seibel NL, O'Brien M, *et al.* Phase I trial of docetaxel administered as a 1-hour infusion in children with refractory solid tumors: a collaborative pediatric branch, National Cancer Institute and Children's Cancer Group trial. *J Clin Oncol* 1997; 15: 1538–43.
23. Blaney SM, Needle MN, Gillespie A, *et al.* Phase II trial of topotecan administered as 72-hour continuous infusion in children with refractory solid tumors: a collaborative Pediatric Branch, National Cancer Institute, and Children's Cancer Group Study. *Clin Cancer Res* 1998; 4: 357–60.
24. Vietti T, Crist W, Ruby E, *et al.* Topotecan window in patients with rhabdomyosarcoma (RMS): an IRSG study. *Proc Am Soc Clin Oncol* 1997; Abstr No 1837.
25. Sparano JA, O'Neill A, Schaefer PL, *et al.* Phase II trial of doxorubicin and docetaxel plus granulocyte colony-stimulating factor in metastatic breast cancer: Eastern Cooperative Oncology Group Study E1196. *J Clin Oncol* 2000; 18: 2369–77.
26. Nabholtz JM, North S, Smylie M, *et al.* Docetaxel (Taxotere) in combination with anthracyclines in the treatment of breast cancer. *Semin Oncol* 2000; 27: 11–8.
27. Salminen E, Bergman M, Huhtala S, Ekholm E. Docetaxel: standard recommended dose of 100 mg/m$^2$ is effective but not feasible for some metastatic breast cancer patients heavily pretreated with chemotherapy-A phase II single-center study. *J Clin Oncol* 1999; 17: 1127.
